# COVID-19 Antibody Testing in Healthcare Workers in Arkansas

**DOI:** 10.7759/cureus.48511

**Published:** 2023-11-08

**Authors:** Manish Joshi, John Theus, Anita Joshi, Matthew Burns, Thaddeus Bartter

**Affiliations:** 1 Medicine, Central Arkansas Veterans Healthcare System, Little Rock, USA; 2 Pathology and Laboratory Medicine, Central Arkansas Veterans Healthcare System, Little Rock, USA; 3 Epidemiology, University of Arkansas for Medical Sciences, Little Rock, USA

**Keywords:** healthcare workers, seroprevalence, sars-cov-2 antibody, covid-19, sars-cov-2

## Abstract

Introduction

Seroprevalence surveys can estimate the cumulative incidence of SARS-CoV-2 infection in a symptom-independent manner, offering valuable data, including herd immunity, that can inform national and local public health policies. To our knowledge, there have been no large studies reporting seroprevalence in healthcare workers (HCWs) in the state of Arkansas. The objective of this study is to measure SARS-CoV-2 seroprevalence in HCWs in a large tertiary-care healthcare system prior to vaccine availability.

Methods

The Central Arkansas Veterans Healthcare System offered SARS-CoV-2 antibody testing prior to the widespread availability of vaccines. After Central Arkansas Veterans Healthcare System institutional review board (IRB) approval had been obtained, a retrospective chart review was used to identify all Central Arkansas Veterans Healthcare System HCWs who had undergone SARS-CoV-2 antibody testing from July 1, 2020, to September 30, 2020. Descriptive analysis was performed using Microsoft Excel (Microsoft Corporation, Redmond, Washington, United States). Correlation and regression tests were performed using SAS 9.4 software (SAS Institute Inc., Cary, NC).

Results

Over the study interval, 170 healthcare personnel had undergone SARS-CoV-2 anti-spike IgG antibody testing. Thirty-seven (21.8%) had positive antibody results. The 37 individuals were mostly women (94.5%), and the average age of the group was 47 years (range 29-69 years). The median antibody titers for those testing positive for antibodies were 10.8 units (range 1.1-58.5). Of the 37 people, 32 had a history of COVID-19 infection proven by reverse transcriptase polymerase chain reaction (RT-PCR).

Conclusion

Serologic testing is feasible for healthcare workers to document an immune response to a prior infection. In this study of HCWs, the rate of positivity among those tested was 21.8%. Data that do not incorporate the cohort of patients with prior infections will underestimate the impact of prior infections on herd immunity statistics and may misinform public policy.

## Introduction

The World Health Organization (WHO) declared coronavirus disease 2019 (COVID-19) a pandemic on March 11, 2020 [1]. Current estimates suggest that a large proportion of the global population has been infected by severe acute respiratory syndrome coronavirus 2 (SARS-CoV-2), predominantly by the omicron variant and its sublineages, which account for 3.8 billion people [1,2]. The capacity to mount an immune response to SARS-CoV-2 has an impact on the duration and severity of illness, and the prevalence of the capacity for an immune response has implications with respect to herd immunity. Antibodies to severe acute respiratory syndrome coronavirus 2, the virus that causes COVID-19, can be detected in the blood of people who have recovered from COVID-19 or people who have been vaccinated against COVID-19. Seroprevalence surveys can estimate the cumulative incidence of SARS-CoV-2 infection in a symptom-independent manner, offering valuable data that can inform national and local public health policies. Although several studies have demonstrated robust long-lasting immunity in people recovered from COVID-19, similar to or better than that induced by current SARS-CoV-2 vaccines [3-14], the contribution of prior infection to seroprevalence has been under-recognized in public policy. Healthcare workers are and have been at the forefront of the COVID-19 response and presumed to be at an elevated risk of illness due to occupational exposure to SARS-CoV-2, in addition to the risks conferred by more typical community-based transmission. The objective of the study is to measure SARS-CoV-2 seroprevalence in healthcare workers (HCWs) in a large tertiary-care healthcare system prior to vaccine availability.

## Materials and methods

The Central Arkansas Veterans Healthcare System offered SARS-CoV-2 antibody testing before the widespread availability of vaccines. After Central Arkansas Veterans Healthcare System institutional review board (IRB) approval (1583463-1) had been obtained, a retrospective chart review was used to identify all Central Arkansas Veterans Healthcare System HCWs who had undergone SARS-CoV-2 antibody testing from July 1, 2020, to September 30, 2020.

Testing had been performed using the FDA-approved Beckman Coulter Access SARS-CoV-2 IgG chemiluminescent immunoassay platform, which detects antibodies to the receptor binding domain of the spike protein. It is an enzyme immunoassay intended for qualitative and semi-quantitative detection of immunoglobulin G (IgG) antibodies to SARS-CoV-2 in plasma using one of the fully automated Access Family of Immunoassay Analyzers. The results of this assay are based on the sample-to-cut-off (S/Co) ratio, and results were reported as reactive (positive), equivocal, or non-reactive (negative) as per the manufacturer’s recommendations based on FDA-approved interpretation criteria.

The charts of patients who had undergone SARS-CoV-2 antibody testing were mined for age, gender, antibody titer if present, and any data concerning prior SARS-CoV-2 infection. Descriptive analysis was performed using Microsoft Excel (Microsoft Corporation, Redmond, Washington, United States). Correlation and regression tests were performed using SAS 9.4 software (SAS Institute Inc., Cary, NC).

## Results

Over the study interval, 170 healthcare personnel had undergone SARS-CoV-2 anti-spike IgG antibody testing (Table 1). Thirty-seven (21.8%) had positive antibody results. The 37 individuals were mostly women (94.5%), and the average age of the group was 47 years (range 29-69 years). The median antibody titers for those testing positive for antibodies were 10.8 units (range 1.1-58.5). Thirty-two of the 37 individuals (86%) had a history of prior COVID-19 infection that had been proven by reverse transcriptase polymerase chain reaction (RT-PCR). The median number of days between COVID PCR positivity and antibody testing was 21 days (range: 11-112 days).

**Table 1:** Demographics and test results of 170 healthcare workers who underwent COVID antibody test

Total healthcare workers tested		170
Positive for antibody		37 (21.8%)
Individuals that tested +ve for COVID antibodies	Median age in years (range)	47 (29 - 69)
	Median titer, unit (range)	10.8 (1.1 - 58.5)
	Number with confirmed prior COVID infection	32 (86.5%)
	Median days since COVID infection for those with confirmed prior infection (range)	21 (11 - 112)

**Figure 1:**
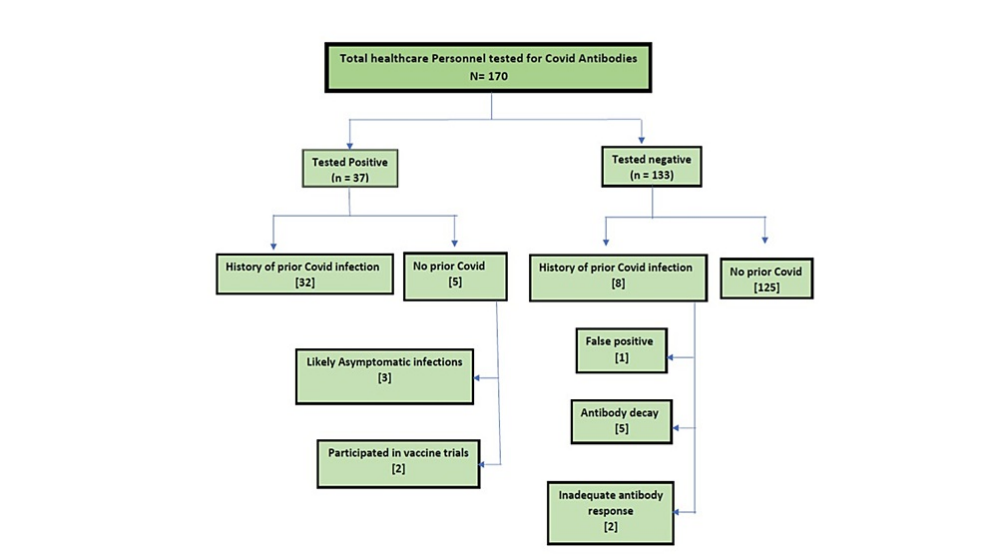
Breakdown of study subjects with respect to antibody status

Five of the 37 patients with positive antibody test results had no history of prior COVID-19 infection (Figure 1). Upon chart review, two were found to have participated in early vaccine trials, the likely cause of antibody production. The remaining three had no history of infection, vaccination, or symptoms and may have had asymptomatic infections. 

Eight of the 133 HCWs who tested negative or equivocal for antibodies had previously tested positive for SARS-CoV-2 by PCR. Five of the eight had borderline titers (>0.5 units), likely due to a >4-month gap between SARS-CoV-2 PCR positivity and antibody testing. Two individuals had been tested <2 weeks after infection and probably had blunted antibody responses such that positive titers were not yet present. One individual was felt upon chart review to have had a false positive SARS-CoV-2 PCR result (and therefore no antibody response). This patient had no consistent symptom history, and the PCR had been performed as a routine screening test.

All but one HCW with a prior COVID-19 infection had recovered at home. One individual had required hospitalization for three days.

**Figure 2:**
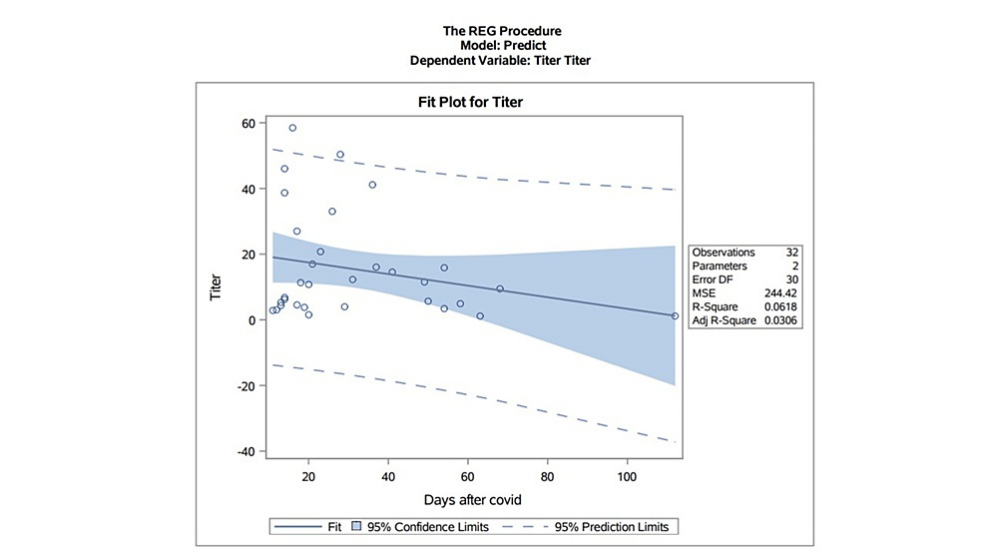
Scatter plot for individuals with antibodies present and proven prior infection

A scatter plot was used to correlate titer levels with time after infection for the subset of cases with both antibody positivity and PCR-proven prior infection (Figure 2). The level of antibody expression soon after infection varied dramatically. Antibody levels waned over time. When antibodies and prior infection were studied, antibody testing lost its sensitivity over time, with a sensitivity of 83.0% four months after infection.

## Discussion

This study raises several points. First, in this study of antibody testing before the widespread availability of SARS-CoV-2 vaccines, 21.8% of the patients who underwent antibody testing were found to have them. Consistent with this is the finding that a chart review of the subjects with positive antibodies identified a definitive prior infection in 86.5%. This was not a random sampling of the population, and thus one cannot extrapolate to the prevalence in the community. The CDC has estimated that, at present, the community seroprevalence related to prior infection is 77.5% [15]. While they do not represent accurate prevalence data, our 21.8% positivity rate in HCWs (in an institution at which personal protective equipment (PPE) was readily available and consistently used) does imply that a significant proportion of persons in the community had already been infected and developed an immune response early in the pandemic.

After widespread vaccination, the antibody response was felt to represent proof of the efficacy of vaccination [16-18]. Much of the country required proof of protection via documentation of vaccination, irrespective of SARS-CoV-2 antibody titers. This ignoring of prior infection as a component of herd immunity vastly underestimates protection in the community.

There is now redundant evidence supporting robust and long-term protection after prior COVID-19 infection [2-14]. COVID-19 infection induces both humoral and cellular responses and generates memory cells, providing durable protection [19-21]. Furthermore, natural SARS-CoV-2 infection induces antibodies against multiple virus epitopes, while the SARS-CoV-2 vaccine produces only S-protein-targeting antibodies [2,20,21]. Natural infection also induces mucosal immunity as a barrier to protection. A recent comprehensive meta-analysis study showed that natural infection induces high levels of protection (>85%) for all major outcomes, including infection, any symptomatic disease, and severe disease [2]. With mutational changes in the virus, protection against reinfection with SARS-CoV-2 wanes over time, but protection against severe disease is maintained at a high level [2].

Figure 2 raises two issues. First, there was a large variation in titers obtained soon after infection. This variation did not imply increased severity of disease; as noted, 36/37 of the patients with positive antibodies had recovered uneventfully at home. Second, and as expected, antibody titers waned with an increasing interval since infection. (Eight of the patients with equivocal or negative antibody results had had a PCR-proven infection.) The half-life of IgG antibodies is about 21 days. These points are consistent with the concept that IgG antibodies are evidence of an orchestrated immune response that includes both humoral and cellular elements. These cells do not continuously generate antibodies in the perpetuum once entrained but are capable of producing recurrent antibody responses if recurrently challenged. While antibodies, when present, do provide evidence of this immune capacity, they do not have quantitative predictive value.

As per the CDC’s interim guidelines for COVID-19, antibody testing not only has clinical utility for patients but also has public health value for evaluating and monitoring population (local, regional, and global) levels of immunity [22]. The current literature suggests that following SARS-CoV-2 infection (irrespective of symptom severity), almost all immunocompetent people develop B and T cell-mediated immunity via triggering antiviral cellular and humoral immune responses, which include neutralizing antibodies against S (which contains two subunits, S1 and S2) and N proteins. The S1 subunit contains a receptor-binding domain and also serves as the main target for neutralizing SARS-CoV-2 antibodies [22]. There are three types of antibodies: IgM, IgG, and IgA-against S and its subunits, which can be detected in serum as early as one week (range one to three) after SARS-CoV-2 infection [22]. Both IgM and IgG antibodies arise and can be detected nearly simultaneously; however, IgG can be detected for a longer duration as IgM antibodies decay more rapidly [22]. It is well known that secretory IgA plays a critical role in protecting against pathogens (at the mucosal surfaces) by neutralizing many known respiratory viruses, including SARS-CoV-2. The clinical significance, however, of measuring serum IgA in SARS-CoV-2 infection is still not completely understood.

There are many FDA-authorized SARS-CoV-2 antibody tests available in the United States. The CDC recommends FDA-authorized tests for both clinical as well as public health purposes. Most of these tests have high sensitivity and specificity, as well as high positive and negative predictive values [22].

Given the lack of perpetuity of antibody generation, antibody testing is inferior to testing for cells that can respond to SARS-CoV-2 antigens if one is trying to define the true prevalence of disease resistance in a community [19-21]. The widespread acceptance of antibody testing for the evaluation of prevalence is probably related to the relative expense and complexity of testing for an immunoactive cell population.

This study has several limitations. Its greatest weakness is that the study was of volunteers and not the result of random sampling, making estimation of true HCW seroprevalence impossible. It is a retrospective chart review study and thus did not allow serial antibody testing that would have more completely defined titer decay over time.

Our study has several strengths. The study was conducted in a large academic tertiary Healthcare Center with dedicated Covid units and adequate availability of personal protective equipment. Data was collected from electronic health records. Dates were exact, thus avoiding recall bias from study participants.

## Conclusions

Seroprevalence surveys are feasible and can estimate the cumulative incidence of SARS-CoV-2 infection in a symptom-independent manner, offering valuable data that can inform national and local public health policies. Although several studies have demonstrated robust, long-lasting immunity in people recovered from COVID-19, similar to or better than that induced by current SARS-CoV-2 vaccines, the contribution of prior infection to seroprevalence has been under-recognized in public policy. It has now been shown specifically for SARS-CoV-2 that prior infection leads to immunoprotection. In this study of HCWs, the rate of positivity among those tested was 21.8%. Data that do not incorporate the cohort of patients with prior infections will underestimate the impact of prior infections on herd immunity statistics and may misinform public policy. We plan to pursue prospective studies using antibody testing to identify seroprevalence at local, regional, and national levels.
